# Correlation of Serum Acylcarnitines with Clinical Presentation and Severity of Coronary Artery Disease

**DOI:** 10.3390/biom12030354

**Published:** 2022-02-23

**Authors:** Olga Deda, Eleftherios Panteris, Thomas Meikopoulos, Olga Begou, Thomai Mouskeftara, Efstratios Karagiannidis, Andreas S. Papazoglou, Georgios Sianos, Georgios Theodoridis, Helen Gika

**Affiliations:** 1 Laboratory of Forensic Medicine and Toxicology, School of Medicine, Aristotle University of Thessaloniki, 54124 Thessaloniki, Greece; mousthom@auth.gr; 2 Biomic_AUTh, CIRI-AUTH Center for Interdisciplinary Research and Innovation, Aristotle University of Thessaloniki, 57001 Thessaloniki, Greece; thomas_meik@hotmail.com (T.M.); olina_18@hotmail.com (O.B.); gtheodor@chem.auth.gr (G.T.); 3 Laboratory of Analytical Chemistry, Department of Chemistry, Aristotle University of Thessaloniki, 57001 Thessaloniki, Greece; 4 First Department of Cardiology, AHEPA University Hospital, Aristotle University of Thessaloniki, St. Kiriakidi 1, 54636 Thessaloniki, Greece; stratoskarag@gmail.com (E.K.); anpapazoglou@yahoo.com (A.S.P.); gsianos@auth.gr (G.S.)

**Keywords:** acylcarnitines, carnitine, coronary artery disease, CAD, cardiovascular disease, CVD, diabetes mellitus, HILIC, LC-MS, metabolic profiling, serum, SYNTAX Score

## Abstract

Recent studies support that acylcarnitines exert a significant role in cardiovascular disease development and progression. The aim of this metabolomics-based study was to investigate the association of serum acylcarnitine levels with coronary artery disease (CAD) severity, as assessed via SYNTAX Score. Within the context of the prospective CorLipid trial (NCT04580173), the levels of 13 circulating acylcarnitines were accurately determined through a newly developed HILIC-MS/MS method in 958 patients undergoing coronary angiography in the AHEPA University Hospital of Thessaloniki, Greece. Patients presenting with acute coronary syndrome had significantly lower median acylcarnitine C8, C10, C16, C18:1 and C18:2 values, compared to patients with chronic coronary syndrome (*p* = 0.012, 0.007, 0.018, 0.011 and <0.001, respectively). Among CAD subgroups, median C5 levels were significantly decreased in unstable angina compared to STEMI (*p* = 0.026), while median C10, C16, C18:1 and C18:2 levels were higher in stable angina compared to STEMI (*p* = 0.019 *p* = 0.012, *p* = 0.013 and *p* < 0.001, respectively). Moreover, median C2, C3, C4 and C8 levels were significantly elevated in patients with diabetes mellitus (*p* < 0.001, <0.001, 0.029 and 0.011, respectively). Moreover, short-chain acylcarnitine C2, C4, C5 and C6 levels were elevated in patients with heavier calcification and lower left ventricular ejection fraction (LVEF) % (all *p*-values less than 0.05). With regard to CAD severity, median C4 and C5 levels were elevated and C16 and C18:2 levels were reduced in the high CAD complexity group with SYNTAX Score > 22 (*p* = 0.002, 0.024, 0.044 and 0.012, respectively), indicating a potential prognostic capability of those metabolites and of the ratio C4/C18:2 for the prediction of CAD severity. In conclusion, serum acylcarnitines could serve as clinically useful biomarkers leading to a more individualized management of patients with CAD, once further clinically oriented metabolomics-based studies provide similar evidence.

## 1. Introduction

Accurate predictors of cardio-metabolic diseases can contribute substantially to their prevention and also provide the possibility for interventions aiming to avoid or delay their onset [1]. Such biomarkers can be timely recognized since metabolic perturbations are present before their clinical outcome. State of the art metabolomics technologies have the ability to monitor subtle changes in the metabolome that occur prior to the phenotypic changes reflecting the disease [2]. These technologies have been recently proved capable of identifying sensitive biomarkers for the early detection or prevention of adverse cardiovascular events. Several metabolomics-based studies have contributed to a better understanding of the metabolic changes that occur in heart failure and ischemic heart disease, and have identified new molecular markers and metabolic signatures of cardiovascular disease (CVD) risk [3,4].

Among the several metabolites identified as potential key drivers of CVD, acylcarnitines, especially those with ≥14 carbon atoms (long-chain acylcarnitines) constitute some of the most commonly implicated metabolites in CVD [5,6]. A number of metabolic profiling studies [7,8,9,10,11,12], where circulating acylcarnitines were measured, support the growing body of evidence of their implication in total CVD risk and prognosis [7,8,10,11,13]. Blood acylcarnitine levels are considered to be characteristic biomarkers describing the composition of cytoplasmic acylcarnitines in many severe pathological conditions such as CAD [11], heart failure [14], type 1 and 2 DM [15], as well as hereditary mitochondrial fatty acid (FA) oxidation disorders derived from genetic etiology [16].

Myocardial metabolism has been long studied. The energy-dependent structure of myocardium contracts incessantly to ensure its optimal function [17], converting chemical energy to mechanical energy [18]. Acylcarnitines seem to play various roles in myocardial metabolism since they are associated with myocardial electrophysiology, contractility, and arrhythmias, as well as with the underlying excitation–contraction coupling modulations [5]. Their crucial role is centered on the transport of long-chain FAs through the mitochondrial membranes for β-oxidation. Mitochondrial FA oxidation in myocytes comprises a high-efficiency energy production route, supplying cardiac muscle with 4 ATP equivalents as well as one acetyl CoA per round of oxidation [19]. Interestingly, in fasting, more than 95% of the produced ATP is derived from FA mitochondrial oxidation in the presence of sufficient oxygen supply [9].

More than 1200 FAs, encountered in the human body, form with L-carnitine and the acylcarnitines, and, therefore, their length differs among different acyl groups [16]. While medium- and short-chain FAs can easily enter the mitochondria, long-chain acyl-CoAs are esterified with carnitine, which acts as a shuttle in order to cross the mitochondrial membrane [6,20]. Apart from the transportation of FAs, acylcarnitines play a regulatory role, crucial for a plethora of intracellular processes for sugar, lipid, and branched-chain amino acids metabolism. They undertake to maintain the mitochondrial acyl-CoA/CoA ratio. They also act as a preventive for the accumulation of acyl-CoA metabolic intermediates in the mitochondria, inhibiting carnitine palmitoyltransferase 1 (CPT1) and, therefore, FA oxidation by their conversion to malonyl-CoA. Furthermore, when the glucagon/insulin ratio is depleted, they stimulate the aerobic oxidation of glucose through the activity of pyruvate dehydrogenase [16].

All the above underline the significant role of acylcarnitines in both global and cardiac-specific metabolism, the disturbances of which underlie most CVDs. Thus, their investigation can provide a better understanding of CVD pathogenesis and contribute to the discovery of novel biomarkers for early CVD diagnosis and prevention [21].

Herein, the investigation of 13 acylcarnitine circulating levels was performed upon their accurate determination by a newly developed LC-MS/MS method in patients admitted to AHEPA University Hospital for clinically indicated coronary angiography, and enrolled in the CorLipid study [22]. The determination of acylcarnitine levels was a part of a series of analyses of CVD-related biomarkers in the context of the CorLipid trial aiming to create a valuable panel for monitoring and predicting future cardiovascular events of patients with coronary artery disease (CAD) [23,24]. For the first time, the severity and the complexity of CAD, as described by the SYNTAX Score (SS), was correlated with circulating metabolic markers such as acylcarnitines, indicating that metabolomics might have brought a paradigm evolving metabolic research.

## 2. Materials and Methods

Acylcarnitine levels were measured in 958 serum samples of patients with CAD, collected under the frame of CorLipid trial (ClinicalTrials.gov Identifier: NCT04580173), a prospective, cross-sectional study performed within the period of July 2019–May 2021. The study cohort included subjects from Northern Greece suffering from acute coronary syndrome (ACS, N = 533) or chronic coronary syndrome (CCS, N = 425), as categorized based on the onset and duration of their symptoms. Patients with ACS were further categorized as: ST-elevation myocardial infarction (STEMI; N = 222), non-ST-elevated myocardial infarction (NSTEMI; N = 170), and unstable angina (UA; N = 141). Hemoglobin A1c (HbA1c) was measured in all 958 patients, and they were stratified as diabetics (N = 316) and non-diabetics (N = 642) according to previous physician-assigned diagnosis or HbA1c levels higher than 6.5%. Similarly, glomerular filtration rate (GFR) was also measured in all participants. Patients with known history of kidney failure, or with GFR lower than 60 mL/min/1.73 m^2^ were considered as having chronic kidney disease (CKD, N = 127). SYNTAX Score (SS) was calculated according to Sianos et al. [25] for all patients and, thereby, they were stratified into 3 SS groups, 0 SS (N = 277), 1–22 SS (N = 471), and >22 SS (N = 210). All trial procedures were in accordance with the WMA Declaration of Helsinki, and all the participants gave written informed consent prior to the execution of coronary angiography, while the study has received the approval of the Scientific Committee of AHEPA University Hospital. Further details on the study can be found in the study protocol [22].

All samples were analysed by a UHPLC-MS/MS method developed and validated by our study group. Hydrophilic Interaction Liquid Chromatography tandem Mass Spectrometry (HILIC-MS/MS) was performed for the quantitation of 13 acylcarnitine analogues, namely Acetyl-L-Carnitine (C2), Propionyl-L-Carnitine (C3), Butyryl-L-Carnitine (C4), Valeryl-L-Carnitine (C5), Hexanoyl-L-Carnitine (C6), Octanoyl-L-Carnitine (C8), Decanoyl-L-Carnitine (C10), Lauroyl-L-Carnitine (C12), Myristoyl-L-Carnitine (C14), Palmitoyl-L-Carnitine (C16), Stearoyl-L-Carnitine (C18), Oleoyl-L-carnitine (C18:1), and Linoleoyl-L-Carnitine (C18:2). Analysis was performed on an Acquity UPLC System (Waters Corporation, Milford, CT, USA) coupled on a XEVO TQD Mass Spectrometer (Waters Corporation, Milford, CT, USA) with electrospray ionization operating in positive mode. Chromatographic separation was carried out on an Acquity BEH Column (2.1 mm × 150 mm, 1.7 m) under isocratic elution conditions. An aliquot of 50 μL of the collected serum samples were treated before their injection into the LC-MS/MS system for analysis. Data acquisition, integration, and identification of all compounds were performed by Waters MassLynx version 4.1 and TargetLynx (Waters). By the applied method, 13 acylcarnitines with a side carbon chain ranging from 2 to 18 were determined with accuracy ranges between 90.4% and 114% and precision within 0.4% and 13.7%. Limit of quantification was at 78.1 ng/mL (384.2 nM) for C2, 2.4 ng/mL (11 nM) for C3, 1.2 ng/mL (5.2 nM) for C4, 1.2 ng/mL (4.9 nM) for C5, 1.2 ng/mL (4.6 nM) for C6, 1.2 ng/mL (4.2 nM) for C8, 1.2 ng/mL (3.8 nM) for C10, 1.2 ng/mL (3.5 nM) for C12, 1.2 ng/mL (3.2 nM) for C14, 2.4 ng/mL (6 nM) for C16, 1.2 ng/mL (2.8 nM) for C18, 2.4 ng/mL (5.6 nM) for C18:1, and 2.4 ng/mL (5.7 nM) for C18:2.

Statistical analysis of the data was performed by using IBM SPSS Statistics for Windows, version 26 (IBM Corp., Armonk, NY, USA). Clinical, procedural, and functional data are presented as median ± 95% confidence intervals CIs (95% CIs) or percentages, as appropriate. Values for all serum metabolites are reported as median ± 95% CIs. Categorical differences between patient groups were evaluated by χ^2^ test for discrete clinical variables. Differences in paired concentrations were evaluated by Wilcoxon signed-rank test. To assess the differences between the study groups, Mann–Whitney U or Kruskal–Wallis test, Bonferroni corrected for multiple comparisons test, were utilized. Spearman’s Rho correlation coefficient was used for correlation analysis. Receiver operating characteristic curve analysis was performed to determine the clinical utility of selected metabolites. Linear regression analysis, using stratified bootstrapping to account for the non-parametric nature of the data, was performed to identify independent predictors of high SYNTAX Score. R, R^2^, and Durbin–Watson metrics along with *p*-values are reported for the linear models. Statistical significance was defined as a value of *p* ≤ 0.05.

## 3. Results

### 3.1. Baseline and Demographic Characteristics

Participants were almost evenly distributed between the age categories with patients under 65 years of age being slightly more than 52.6% of the total population (N = 504). Only 26.6% were female and 58.5% (N = 560) were hypertensive along with 37.9% being dyslipidemic. A large percentage of CorLipid participants (44.2%) reported current use of tobacco products at enrollment. Baseline clinical and demographic characteristics of the study participants, briefly described in Table 1 and Table 2, share significant similarities with relevant cohorts recently published [26,27,28].

### 3.2. Serum Acylcarnitines Concentrations

Median concentrations of acylcarnitines in the studied population were determined at 2615 ng/mL (12.9 μM) for C2, 146.7 ng/mL (0.68 μM) for C3, 34.6 ng/mL (0.15 μM) for C4, 23.7 ng/mL (0.08 μM) for C5, 25.4 ng/mL (0.10 μM) for C6, 49.2 ng/mL (0.17 μM) for C8, 81.4 ng/mL (0.26 μM) for C10, 24.9 ng/mL (0.07 μM) for C12, 16.3 ng/mL (0.04 μM) for C14, 55.1 ng/mL (0.14 μM) for C16, 17.6 ng/mL (0.04 μM) for C18, 78.0 ng/mL (0.18 μM) for C18:1, and 49.2 ng/mL (0.12 μM) for C18:2. Blood acylcarnitine levels were in agreement with previous studies [8,11,12,29], ranging in a common concentration span and order of magnitude.

The evaluation of the obtained serum acylcarnitine concentrations was performed in patients categorized to characteristic CAD-related groups according to their clinical data. At first, patients were categorized into two main CAD groups, ACS and CCS. Patients with ACS were further stratified into STEMI, NSTEMI, and UA. The comorbidity of diabetes mellitus (DM) was also evaluated for each CAD category since it constitutes a highly prevalent comorbidity with a heightened risk of adverse outcomes in CAD [30]. Finally, the correlation of circulating acylcarnitine levels with the calculated SYNTAX Score was performed as an indicator of the correlation between the complexity of CAD and its metabolic imprint.

### 3.3. Acylcarnitine Levels in ACS vs. CCS Patients

Based on the clinical data, the patients were first classified into acute (ACS) and chronic coronary syndrome (CCS). The median concentrations of all measured acylcarnitines and 95% CIs, as well as the relevant AUC values for ACS and CCS group of patients are provided in the Table S1.

Based on the determined concentrations, ACS patients demonstrated significantly lower median values of some acylcarnitine levels compared to CCS patients. As seen in Table 3, C8 (*p* = 0.012), C10 (*p* = 0.007), C16 (*p* = 0.018), C18:1 (*p* = 0.011), and C18:2 (*p* < 0.001) presented lower values in ACS compared to CCS patients. Additional graphical illustrations, indicating the differentiation of these five acylcarnitines in ACS vs. CCS patients, are presented in the Supplementary Materials. Box plots of acylcarnitines C8, C10, C16, C18:1, and C18:2 median levels for both ACS and CCS patients are illustrated in Figure S1A, while their respective ROC curves with the best AUC value (AUC value = 0.576, 95% CI 0.539–0.612, *p*  <  0.001) are those of C18:2, presented in Figure S1B.

Diabetes mellitus is a severe comorbidity in CAD patients, and its association with acylcarnitine levels was explored in our population. Patients with either known DM history or HBA1C > 6.5 (N = 316) were considered as diabetics [12,31,32].

Based on the determined concentrations, several acylcarnitine levels in DM patients were found to be elevated compared to those of non-DM patients, independently of CAD status (N = 642). These were C2 (*p* < 0.001), C3 (*p* < 0.001), C4 (*p* = 0.029), and C8 (*p* = 0.011) (see Figure S2).

By comparing acylcarnitine levels of ACS with CCS patients while considering their DM history, C8 (*p* = 0.008), C10 (*p* = 0.004), C12 (*p* = 0.026), C14 (*p* = 0.011), C16 (*p* = 0.012), C18 (*p* = 0.038), C18:1 (*p* = 0.003), and C18:2 (*p* < 0.001) demonstrated lower median values in non-DM CCS patients; none of these, however, were significantly differentiated in DM patients of either groups. In Table 4, median values of all measured acylcarnitines levels are presented for ACS and CCS patients with and without DM history.

Regardless of the ACS status, ROC analysis indicated that C2 had the highest discriminatory power 0.591 (95% CIs 0.553–0.629, *p*  <  0.001) (see Table S3). Notably, ROC analysis of the discriminant C8, C10, C16, C18:1, and C18:2 showed that AUC values were improved, especially for C18:2 from 0.575 to 0.593 (95% CIs 0.549–0.638, *p*  <  0.001) for ACS patients when their DM history was considered (Table S4 and Figure S3).

### 3.4. Acylcarnitine Levels in CAD Subgroups

The patients were classified according to clinical, electrocardiographic, and laboratory parameters into the following CAD subgroups: STEMI, NSTEMI, UA, and SA, aiming to assess the diagnostic capability of acylcarnitines for each CAD subgroup.

**Figure 1:**
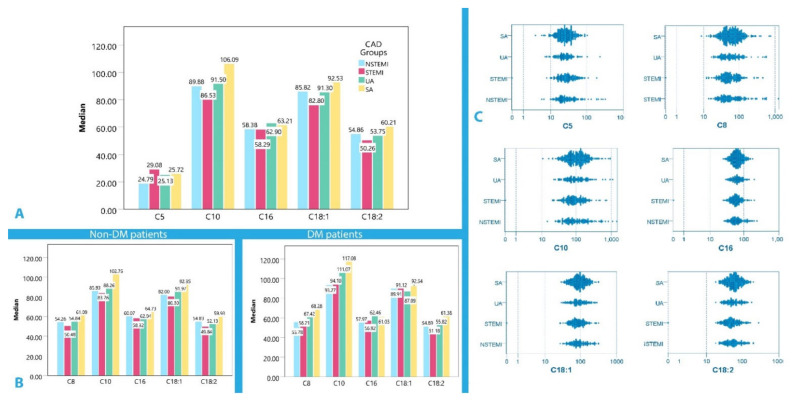
(**A**) Box plot with the respective medians for each of the important acylcarnitines for the CAD groups, (**B**) Box plot with the respective medians for each of the important acylcarnitines for the CAD groups in DM and non-DM patients, (**C**) ACS vs. CCS 2-D log scaled plots for C5, C8, C10, C16, C18:1, and C18:2 level distributions.

Comparison of serum acylcarnitine levels among the different CAD subgroups (STEMI, NSTEMI, UA, and SA) demonstrated that C5 median levels were decreased in UA in comparison with STEMI (*p* = 0.026). Moreover, median C10, C16, C18:1, and C18:2 levels were higher in SA compared to STEMI (*p* = 0.019 *p* = 0.012, *p* = 0.013, and *p* < 0.001, respectively), as seen in Table 5. The stacked histogram with the respective medians for each of the significant acylcarnitines according to the clinical presentation of CAD is depicted in Figure 1A, whereas log scaled 2-D dot plots of comparative distributions are seen in Figure 1C.

When DM history was taken into account, acylcarnitine levels were similarly affected in all CAD groups, with the only exception being C8, which was differentiated between STEMI and SA in non-DM patients (*p* = 0.045). Furthermore, DM patients, regardless of CAD category, did not present any discerning differences in median acylcarnitine levels as they were all similarly elevated (see Figure 1B, Table S5).

### 3.5. Correlation of SYNTAX Score with Acylcarnitine Levels in Patients with ACS

Serum acylcarnitine levels were categorized according to the SYNTAX Score (SS) groups: 0 SS, 1–22 SS, and >22 SS, and the relevant outcomes are presented in Table S6.

**Figure 2:**
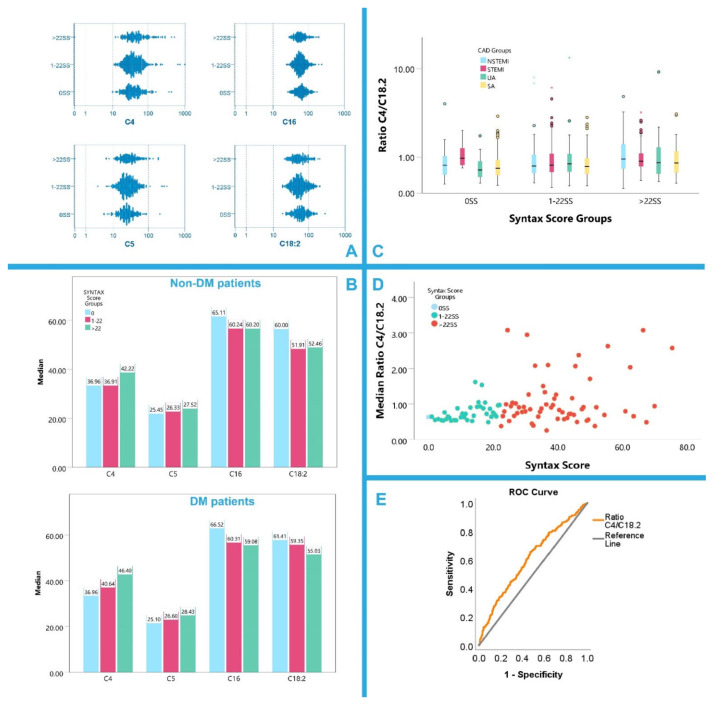
(**A**) SS Groups, log10 scaled 2-D plots for C4, C5, C16, and C18:2 level distributions. (**B**) Bar plots with the respective medians for each of the important acylcarnitines for the SS groups. (**C**) Log10 scaled grouped box plots with the respective medians for the C4/C18:2 carnitine ratio in each of the CAD groups for all three SS groups. (**D**) Linear relationship of the C4/C18:2 ratio to the continuous SS as identified by the linear regression model presented in text. (**E**) ROC analysis of the C4/C18:2 ratio for high risk SS Group > 22.

Based on the obtained results, it was found that C4 levels were elevated in the >22 SS group compared to the minimal risk group 0 SS (*p* = 0.002) and to the medium risk group 1–22 SS, (*p* = 0.005). Similarly, C5 levels were elevated in the >22 SS group compared to the minimal risk group 0 SS (*p* = 0.024). On the contrary, C16 and C18:2 levels demonstrated a reverse relationship to the SS, indicating a potential prognostic capability as biomarkers for the prediction of CAD severity. C16 and C18:2 levels were increased in the 0 SS group compared to the other two groups (C16, *p* = 0.031; C18:2, *p* = 0.019 for 1–22 SS and C16, *p* = 0.044; C18:2, *p* = 0.012 for >22 SS, respectively). Statistically significant correlations between acylcarnitine levels and SS groups are presented in Table 6. In Figure 2A, these data are graphically illustrated in log10 scaled 2-D plots.

With regards to DM and SS, it was found that in non-DM patients, only acylcarnitine C18:2 levels were significantly differentiated among the groups of 0 SS vs. 1–22 SS and 0 SS vs. >22 SS, (*p* = 0.002 and *p* = 0.032, respectively, for the paired comparisons) with the levels of this long-chain acylcarnitine being higher in patients with 0 SS than the other two SS groups, indicating its use as a potential positive prognostic biomarker. On the other hand, short-chain acylcarnitines C4 and C5 were differentiated in DM group. The levels of C4 and C5 were higher in the >22 SS group than in the 0 SS group with *p* = 0.007 and *p* = 0.040, respectively, for the paired comparisons, indicating their potential use as negative CVD risk biomarkers. Statistically significant acylcarnitine data in DM and non-DM patients based on their SS are depicted in Figure 2B.

As well as the SYNTAX Score, clinical metrics such as heavy calcification and LVEF% may present biological relevance with acylcarnitine levels. Short-chain acylcarnitine C2, C4, C5, and C6 levels were elevated in patients with heavy calcification and low LVEF%, indicating a potential connection of these specific acylcarnitines with these metrics of severe CAD and left ventricular systolic dysfunction (see Table S7).

Acylcarnitine C4 and C18:2 levels had the most significant differentiations among the different SS groups, thus, a ratio of C4/C18:2 could be proposed as a useful marker of CAD severity. While acylcarnitines might be highly correlated and prone to confounding effects, the ratio C4/C18:2 was not correlated to most descriptive parameters.

A stratified bootstrapped linear regression model (R = 0.404, R^2^ = 0.164, Durbin–Watson 1.566, *p* = 0.001) adjusted for age, sex, smoking, DM, statin use, and CAD groups showed the proposed ratio of C4/C18:2 to be an independent predictor of higher SS with B = 2.010 ±0.613 (95%CIs 1.159–3.587, *p* = 0.002). However, other acylcarnitine ratios tested did not yield notable results. Figure 2C shows the different C4/C18:2 ratios across the SS groups. Detailed results are presented in Table S8. Figure 2D illustrates the linear relationship of the C4/C18:2 ratio with the SS. Its AUC value of 0.607 (*p* < 0.001, 95%CIs 0.563–0.651) shown in Figure 2E was indicative of its potential to identify high risk patients categorized by an anatomical metric, such as the SS. Another ratio between C2 and C16 was also considered, but it showed a weaker AUC value of 0.597 (*p* < 0.001, 95%CIs 0.547–0.638) and did not demonstrate a significant predictive role in a similar linear regression model for SS prediction.

### 3.6. Acylcarnitne Levels in Chronic Kidney Disease

All acylcarnitines were statistically higher in CKD compared to the rest of the population, as seen in Table S9. Acylcarnitines C2, C3, C4, C5, C6, C8, C10, C12, C14, C16, C18, C18:1, and C18:2 were all elevated, revealing the critical role of comorbidities in patients’ metabolic profile. Figure S4 illustrates the direct distribution comparisons and the mean value of each measured acylcarnitine.

## 4. Discussion

Narrowing or occlusion of the coronary arteries due to plaque formation and, consequently, impaired oxygen supply lead to perturbations in systemic and myocardial metabolism [33]. Broadening the knowledge of the cascade of events attributed to the etiopathogenesis of this complex chronic inflammatory disease is a determinant factor in the fight against its development and progression [13].

The CorLipid study aimed to create an integrated panel of CAD-related biomarkers with the potential to support clinical decision making for the effective stratification and management of CAD patients [22]. Previous publication on a subset of STEMI patients enrolled in the CorLipid trial has revealed the prognostic value of serum ceramide levels on the occurrence of large thrombus burden [24]. Herein, we discuss the results from the quantitative profiling of 13 serum acylcarnitines as acquired by a state of the art LC-MS/MS method in 958 patients with CAD.

Regarding the role of acylcarnitines in CAD pathophysiology, this is still under investigation. However, experimental studies seem to associate acylcarnitines with macrophage FA catabolism monitoring [34]. Serum acylcarnitine analysis can quantify the state of β oxidation, which has been closely linked with vascular inflammation in several studies. The accumulation of acylcarnitine intermediates in extracellular fluid is derived from the inefficient β-oxidation and altered mitochondrial metabolism, particularly in advanced age, leading to impaired FA recycling [6,34]. Furthermore, the ageing process is also related to increased mitochondrial production of reactive oxygen species resulting in augmented vascular inflammation and affecting the plaque composition and rupture [6]. In clinical studies, acylcarnitines have recently been associated with increased risk of atherosclerotic plaque formation, independently of traditional CV risk factors [35].

In our study, the statistical analysis of the obtained dataset of 958 serum samples managed to distinguish ACS from CCS patients based on the measured acylcarnitine levels. More specifically, medium- and long-chain acylcarnitines, namely C8, C10, C16, C18:1, and C18:2 were differentiated in patients who suffered from ACS compared to those with CCS, regardless of their comorbidities. Octadecadienylcarnitine proved to be the strongest discriminator of the two groups. Interestingly, all of those acylcarnitines were elevated in CCS. This finding is difficult to interpret since this was not a case-control study and all participants suffered from CAD derived from an atherosclerosis-related pathophysiological mechanism, despite the different clinical manifestation.

From the broad previous categorization, patients were further categorized upon CAD manifestation into four more coherent groups including NSTEMI, STEMI, UA, and SA. Accumulation of a short-chain acylcarnitine (C5) and depletion of medium-chain acylcarnitine (C10) were observed in STEMI patients compared to patients with UA and SA, respectively. Long-chain acylcarnitines C16, C18:1, and C18:2, which are used as shuttles for the most common dietary FAs to cross the mitochondrial membrane, were higher in SA compared to STEMI. Considering the necessity of long-chain FAs to deliver a carnitine shuttle, the elevation of palmitoylcarnitine could probably suggest either a burdened and dysfunctional mitochondrial transport or a perturbed FA oxidation [11].

Previous studies on circulating acylcarnitines did not yield a consistent finding, presenting various trends of the different short-, medium- and long-chain analogues in correlation to CVD. In a prospective study of 4164 patients with SA, the amount of five serum acylcarnitines including C2 (acetyl-), C3 (propionyl-), C5 (isovaleryl-), C8 (octanoyl-), and C16 (palmitoyl-) carnitine were evaluated, and it was shown that C2, C8, and C16 were associated with an increased risk of CV death independently of established hallmarks such as troponin T. Perturbed FA metabolism affected directly octanoyl- and palmitoyl carnitine levels, while propionyl- and (iso)valeryl carnitine levels could also reflect branched-chain amino acids metabolism [11].

Rizza et al. aimed to quantify free carnitine (C0) and 30 acylcarnitines among other metabolites in a group of elderly individuals with highly prevalent CAD history. The study indicated that serum medium- and long-chain acylcarnitines were independently linked with a higher incidence of CV events [36]. The findings of a case-control study in which STEMI, NSTEMI patients, and patients with chest pain accompanied with abnormal troponin T levels were enrolled, revealed elevation of short- and medium-chain acylcarnitines levels compared to healthy individuals. Among long-chain acylcarnitines, only C16:1 was significantly altered (increased) in the diseased group. Furthermore, the ratio of free carnitine to the sum of short- or medium-chain acylcarnitines was decreased in all patient groups [8].

In another study where the levels of 45 acylcarnitines were measured in plasma from arterial blood on 2023 patients with suspected CAD undergoing coronary angiography, medium-chain acylcarnitines, short- and long-chain dicarboxylacylcarnitines were found to independently predict the risk of CV events. Dicarboxylic acids were derived either from FA ω-oxidation and cytochrome p450 enzymatic activity being shortened via *β*-oxidation, or from CoA esters though amino acids oxidation [37]. These results pointing to different directions and conclusions may indicate that various comorbidities—probably metabolic—may exert a significant confounding effect that has to be taken into account.

Several CAD-related comorbidities of the CorLipid participants were evaluated in the frame of acylcarnitine profiling. Patients with type 2 DM are known to be at a higher risk of developing CAD and, remarkably, most of those patients finally die from CVD, including CAD [38]. Based on our data, diabetic patients’ serum samples seem to have higher levels of several acylcarnitines, independently of the clinical presentation of CAD. In contrast, the non-diabetic population presented fluctuations of medium- and long-chain acylcarnitines depending on the presence of ACS or CCS. This finding may indicate the level of DM interference with acylcarnitine profiles in the context of CAD. In the same manner, considering DM, regardless of CAD manifestation groups, acylcarnitines were elevated indicating a lower discriminatory significance. However, in non-DM patients, C5 lost its statistical significance that it has previously shown, while octanoyl-carnitine (C8) revealed a remarkable discriminatory power between STEMI and SA.

Recently, the levels of plasma C2, C4, C6, C8, C10, and C12 were linked to increased CVD risk in a cross-sectional study of Chinese patients with type 2 DM in which dry blood spot samples were collected by finger puncture after 8 h fasting and 25 acylcarnitines were measured by a Q TRAP MS method [12]. As reported by the authors, increased catabolism of long-chain FAs could inhibit short- and medium-chain FA oxidation, resulting in the accumulation of acyl-CoA and short- and medium-chain acylcarnitines [12].

Moreover, according to our study, participants who suffered from CKD demonstrated increased acylcarnitine levels compared to the rest of the cohort population. Similar findings have been previously reported. Kalim et al. suggested that, among 18 other acylcarnitines measured by an LC-MS method, elevated oleoyl-carnitine (C18:1) could be used as a predictor of 1-year CV mortality in end stage renal disease patients [7]. Chronic hemodialysis could be responsible for the diminished renal L-carnitine biosynthesis and renal short-chain acylcarnitines excretion [7].

Regarding correlations of traditional CVD-related biochemical parameters, the existing literature has shown that acylcarnitine levels may be positively correlated with BMI [11,12], plasma glucose, Apo B, and CRP levels [11], and negatively correlated with LVEF% as well, [14]. Based on our results, short-chain acylcarnitine levels were elevated in patients with low LVEF%, indicating their association with decreased systolic function and potently with increased heart failure incidence. However, no significant correlation was observed between acylcarnitine levels and BMI. Finally, the ratio of ApoB/ApoA1, which indicates the existing balance between atherogenic and anti-atherogenic lipoproteins [39], was significantly correlated to C16, C18, C18:1, and C18:2 levels in NSTEMI, supporting their significant metabolic implications.

Furthermore, based on our findings, acylcarnitine levels were significantly associated with the SS, bridging, thereby, the angiographic complexity of CAD with these circulating metabolic biomarkers. To our knowledge, this is the first study attempting to find a dynamic relationship of acylcarnitines with the severity of CAD, as described by the SS. Among the studied acylcarnitine ratios, C4/C18:2 may serve as an independent predictor of SS based on its statistically significant and adequate AUC value. The levels of short-chain acylcarnitines, C4 and C5, were found to be elevated in high risk patients based on SS. In contrast, long-chain acylcarnitines C16 and C18:2 were decreased in those patients compared to lower risk patients. Interpreting the acylcarnitine reverse relationship to SS is not an easy task and may be linked to fundamental and complex events of the development of plaques and progressively to the different clinical outcomes.

Our study provided some novel findings, adding further evidence on the significance of serum acylcarnitines for the prediction of CAD progression. It should be mentioned, however, that several limitations might be considered. The CorLipid study is a cross-sectional, single center study, and the generalization and extrapolation of its findings to other populations may be complicated. All participants underwent clinically indicated coronary angiography and, thus, the interpretation of acylcarnitine levels fluctuations should have been attributed to different clinical manifestations of CAD, which can be more demanding than in case-control studies. Moreover, the comparisons of serum acylcarnitine levels among different study subgroups have not been adjusted for potential confounders, with the exception of our primary analysis on the prediction of the SS, where we conducted multivariate linear regression modeling. Furthermore, data on the dietary habits and feeding history of our study participants were not available for all of them since a small proportion of high risk patients underwent urgent coronary angiography at the time of their arrival at the emergency department. This could probably be a confounder for our analyses since in fasting acylcarnitines in myocardium are approximately 5-fold higher than in fed state [9]. Strand et al. demonstrated that fasting acylcarnitine levels could be possibly more accurate for the prediction of major adverse CV events since they are less affected by nutritional factors [11]. Finally, we should also state as a potential limitation that the AUC of 0.6 drawn for the prediction of the SS based on the acylcarnitine ratio C4/C18.2, although significant, is relatively weak. Hence, further studies are warranted prior to reaching definite conclusions for acylcarnitine utility as a biomarker.

## 5. Conclusions

In conclusion, the findings of this acylcarnitine profiling study add further evidence to the existing literature regarding the role of acylcarnitines in the development and severity of CAD. A short-chain to long-chain acylcarnitine (C4/C18:2) ratio was found to be independently correlated with higher SS. Such outcomes might join together the complex pathophysiology of CAD and the affected myocardial metabolism. Although substantial efforts are still required for our LC-MS/MS-based acylcarnitine measurements to pass to direct clinical translation, similar studies could pave the way for personalized medicine and individualized CAD patient management through the discovery of patient-specific metabolic fingerprints.

## Figures and Tables

**Table 1:** Baseline clinical and demographic characteristics of the studied population.

	Sex						CAD				
Baseline Characteristics		Female		Male			CCS		ACS		
		N	N%	N	N%	# *p*	N	N%	N	N%	# *p*
**Hypertension**	**No**	84	21.10	314	78.90	0.0001	149	37.40	249	62.60	0.0001
	**Yes**	171	30.50	389	69.50		276	49.30	284	50.70	
**Diabetes Mellitus**	**No**	161	25.10	481	74.90	0.124	285	44.40	357	55.60	0.979
	**Yes**	94	29.70	222	70.30		140	44.30	176	55.70	
**Dyslipidemia**	**No**	145	24.40	449	75.60	0.046	232	39.10	362	60.90	0.0001
	**Yes**	110	30.30	253	69.70		193	53.20	170	46.80	
**Smoking**	**No**	190	35.50	345	64.50	0.0001	286	53.50	249	46.50	0.0001
	**Yes**	65	15.40	358	84.60		139	32.90	284	67.10	
**Age groups**	**65<**	99	19.60	405	80.40	0.0001	201	39.90	303	60.10	0.003
	**65>**	155	34.30	297	65.70		224	49.60	228	50.40	
**Chronic Kidney Disease**	**No**	196	24.00	622	76.00	0.0001	374	45.70	444	54.30	0.005
	**Yes**	55	43.30	72	56.70		41	32.30	86	67.70	
**SYNTAX Score Groups**	**0**	103	37.20	174	62.80	0.001	192	69.30	85	30.70	0.0001
	**1 to 22**	99	21.00	372	79.00		167	35.50	304	64.50	
	**>22**	53	25.20	157	74.80		66	31.40	144	68.60	
**CAD Groups**											
**Baseline Characteristics**		**NSTEMI(α)**		**STEMI(β)**		**UA (γ)**		**SA(δ)**			
		**N**	**N%**	**N**	**N%**	**N**	**N%**	**N**	**N%**		* *p* (pair)
**Hypertension**	**No**	63	15.80	129	32.40	57	14.30	149	37.40		0.005 (β–α), <0.001 (β–γ), <0.001 (β–δ),
	**Yes**	107	19.10	93	16.60	84	15.00	276	49.30		
**Diabetes Mellitus**	**No**	111	17.30	160	24.90	86	13.40	285	44.40		0.164
	**Yes**	59	18.70	62	19.60	55	17.40	140	44.30		
**Dyslipidemia**	**No**	104	17.50	166	27.90	92	15.50	232	39.10		0.045 (β–α), >0.001 (β–δ),
	**Yes**	65	17.90	56	15.40	49	13.50	193	53.20		
**Smoking**	**No**	78	14.60	94	17.60	77	14.40	286	53.50		>0.001(δ–α), >0.001(δ–β)
	**Yes**	92	21.70	128	30.30	64	15.10	139	32.90		
**Age groups**	**65<**	93	18.50	143	28.40	67	13.30	201	39.90		0.013 (β–γ), >0.001 (β–δ),
	**65>**	76	16.80	79	17.50	73	16.20	224	49.60		
**Chronic Kidney Disease**	**No**	132	16.10	191	23.30	121	14.80	374	45.70		<0.001 (δ–α)
	**Yes**	38	29.90	29	22.80	19	15.00	41	32.30		
**SYNTAX Score Groups**	**0**	25	9.00	11	4.00	49	17.70	192	69.30		<0.001 (δ–α), <0.001 (δ–β), <0.001 (γ–α), <0.001 (γ–β)
	**1 to 22**	90	19.10	151	32.10	63	13.40	167	35.50		
	**>22**	55	26.20	60	28.60	29	13.80	66	31.40		

# Mann–Whitney U test, * Kruskal–Wallis test Bonferroni corrected.

**Table 2:** Baseline biochemical characteristics of the studied population.

	Sex							CAD						
	Female			Male				CCS			ACS			
	Median	↓95.0% CIs	↑95.0% CIs	Median	↓95.0% CIs	↑95.0% CIs	# *p*-Value	Median	↓95.0% CIs	↑95.0% CIs	Median	↓95.0% CIs	↑95.0% CIs	^#^ *p*
**BMI**	28.1	27.5	28.8	27.9	27.7	28.4	0.407	28.27	27.8	28.7	27.8	27.5	28.4	0.127
**CHOL**	163	155	171	158	154	163	0.073	160	156	166	158	154	165	0.96
**TG**	127	119	133	124	120	130	0.891	122	116	128	129	122	135	0.039
**HDL**	45	42	46	39	39	41	0	43	42	46	39	39	41	0
**LDL**	87	81	94	89	86	93	0.91	87	83	92	89	85	94	0.104
**TnThs**	27	20	41	41	30	52	0.003	14	13	16	250	180	357	0
**LVEF (%)**	0.55	0.55	0.60	0.55	0.55	0.60	0.121	0.60	0.60	0.65	0.50	0.50	0.55	0
**GFR**	83.6	78.7	88	96.6	94	99.3	0	89.2	86.3	93.6	95.8	92.4	98.7	0.389
	**CAD Groups**													
	**NSTEMI (α)**			**STEMI (β)**			**UA (γ)**			**SA (δ)**				
	**Median**	**↓95.0% CIs**	**↑95.0% CIs**	**Median**	**↓95.0% CIs**	**↑95.0% CIs**	**Median**	**↓95.0% CIs**	**↑95.0% CIs**	**Median**	**↓95.0% CIs**	**↑95.0% CIs**	* p **(pair)**	
**BMI**	27.7	27	28.4	28.1	27.7	28.7	27.7	26.7	29	28.27	27.8	28.7	0.189	
**CHOL**	155	148	169	162	154	169	155	151	167	160	156	166	0.648	
**TG**	130	120	142	125	116	136	134	117	142	122	116	128	0.159	
**HDL**	38	37	40	38	36	40	40	38	43	43	42	46	>0.001 (β–δ), >0.001 (α–δ)	
**LDL**	85	78	101	94	90	106	86	81	92	87	83	92	0.024 (γ–β)	
**TnThs**	244	175	347	1409	1175	1915	18	16	26	14	13	16	>0.001 (δ–α), >0.001 (δ–β), >0.001 (δ–γ,) >0.001 (γ–α), >0.001 (γ–β), >0.001 (α–β)	
**LVEF (%)**	0.50	0.50	0.55	0.45	0.45	0.50	0.55	0.55	0.60	0.60	0.60	0.65	>0.001 (δ–α), >0.001 (δ–β), 0.015 (γ–α), >0.001 (γ–β), >0.001 (α–β),	
**GFR**	91.8	84.4	98.3	98.1	92.8	101.4	96.1	88.5	100	89.2	86.3	93.6	0.086	

^#^ Mann–Whitney U test, * Kruskal–Wallis test Bonferroni corrected, BMI: Body Mass Index, CHOL: Cholesterol, TG: Triglycerides, HDL: high-density lipoprotein LDL: low-density lipoprotein, TnThs: high sensitive cardiac troponin, LVEF: left ventricular ejection fraction.

**Table 3:** ACS and CCS patient median level values (μg/L) of acylcarnitines C8, C10, C16, C18:1, and C18:2.

	CAD						
	CCS			ACS			
	Median	↓95.0% CIs	↑95.0% CIs	Median	↓95.0% CIs	↑95.0% CIs	* p
C8	63.06	58.68	68.55	54.75	51.21	57.68	0.012
C10	106.12	96.74	116.37	88.51	83.51	93.92	0.007
C16	63.21	60.85	65.63	59.97	57.66	61.97	0.018
C18:1	92.54	88.15	97.76	84.43	80.05	89.91	0.011
C18:2	60.22	57.89	63.29	51.89	50.44	54.67	<0.001

* Mann–Whitney U test.

**Table 4:** DM vs non-DM acylcarnitines levels (μg/L) in ACS and CCS patients. Statistically significant values are shown in bold.

		Non-DM Patients				DM Patients (HBA1C > 6.5)			
		Median	↓95.0% CIs	↑95.0% CIs	* *p*	Median	↓95.0% CIs	↑95.0% CIs	* *p*
C2	CCS	2957.11	2734.56	3089.26	0.321	2761.05	2529.49	2964.76	0.088
	ACS	3036.99	2817.51	3365.70		3289.10	2976.97	3801.96	
C3	CCS	172.44	162.80	184.61	0.979	166.81	155.35	177.16	0.310
	ACS	185.16	172.28	203.39		191.23	177.00	214.25	
C4	CCS	37.76	34.94	40.46	0.597	37.21	35.39	40.48	0.876
	ACS	42.47	37.42	48.06		40.62	37.34	45.16	
C5	CCS	25.91	24.91	28.07	0.463	26.17	24.95	28.33	0.351
	ACS	25.25	24.02	29.21		26.81	24.57	30.87	
C6	CCS	29.84	28.47	31.34	0.078	27.32	25.44	29.10	0.522
	ACS	30.35	28.07	33.39		30.47	28.46	33.44	
**C8**	**CCS**	**61.50**	**56.90**	**67.91**	**0.008**	52.59	49.35	55.94	0.573
	**ACS**	**67.82**	**56.94**	**77.31**		59.00	53.41	65.99	
**C10**	**CCS**	**103.06**	**95.47**	**112.23**	**0.004**	84.57	79.88	91.77	0.612
	**ACS**	**117.07**	**92.22**	**132.23**		97.62	86.47	115.32	
**C12**	**CCS**	**29.95**	**27.55**	**31.44**	**0.026**	26.48	24.49	28.74	0.995
	**ACS**	**29.88**	**27.29**	**33.30**		29.71	27.15	32.21	
**C14**	**CCS**	**19.26**	**18.12**	**19.86**	**0.011**	17.54	16.81	18.74	0.715
	**ACS**	**19.05**	**17.91**	**20.53**		18.90	17.62	20.53	
**C16**	**CCS**	**64.79**	**61.80**	**66.64**	**0.012**	60.15	57.23	62.77	0.578
	**ACS**	**61.00**	**58.42**	**65.28**		59.54	56.19	63.82	
**C18**	**CCS**	**19.22**	**18.19**	**19.91**	**0.038**	18.11	17.49	18.77	0.758
	**ACS**	**18.74**	**17.75**	**19.77**		18.76	17.63	19.32	
**C18:1**	**CCS**	**93.41**	**87.84**	**99.43**	**0.003**	82.36	77.78	88.15	0.813
	**ACS**	**92.53**	**86.95**	**100.00**		89.91	81.61	97.51	
**C18:2**	**CCS**	**59.96**	**56.67**	**63.31**	**<0.001**	50.89	49.07	53.70	0.202
	**ACS**	**61.35**	**57.75**	**67.58**		54.99	51.18	60.46	

* Mann–Whitney U.

**Table 5:** Acylcarnitines C5, C10, C16, C18:1, and C18:2 levels (μg/L) with statistical significance according to the clinical presentation of CAD.

	CAD Groups												
	NSTEMI (α)			STEMI (β)			UA (γ)			SA (δ)			
	Median	↓95.0% CIs	↑95.0% CIs	Median	↓95.0% CIs	↑95.0% CIs	Median	↓95.0% CIs	↑95.0% CIs	Median	↓95.0% CIs	↑95.0% CIs	* p (Pair)
**C5**	24.79	23.46	28.80	29.08	26.36	30.73	25.13	22.86	27.70	25.72	24.95	27.50	0.026 (δ–γ)
**C10**	89.88	78.25	105.42	86.53	79.36	94.45	91.50	83.49	110.42	106.09	96.74	116.37	0.019 (δ–β)
**C16**	58.38	55.03	63.64	58.29	55.82	60.89	62.90	60.82	66.52	63.21	60.85	65.63	0.012 (δ–β)
**C18:1**	85.82	78.64	94.91	82.80	76.43	88.61	91.30	79.31	97.06	92.53	88.15	97.76	0.013 (δ–β)
**C18:2**	54.86	50.74	59.54	50.26	47.34	52.41	53.75	50.00	60.48	60.21	57.89	63.29	>0.001 (δ–β)

* Kruskal–Wallis (pair) Bonferroni corrected.

**Table 6:** Acylcarnitines with significantly different levels (μg/L) across SYNTAX Score groups.

SYNTAX Score Groups										
	0 (a)			1 to 22 (b)			>22 (c)			
	Median	↓95.0% CIs	↑95.0% CIs	Median	↓95.0% CIs	↑95.0% CIs	Median	↓95.0% CIs	↑95.0% CIs	* p (Pair)
**C4**	36.96	34.21	40.46	37.95	35.6	40.18	45.16	38.94	49.61	0.002 (a–c) 0.005 (b–c)
**C5**	25.25	23.99	26.36	26.41	24.95	28.63	27.82	25.34	30.79	0.024 (a–c)
**C16**	65.18	62.57	67.9	60.28	57.95	62.48	59.27	56.28	61.94	0.031 (c–a) 0.044 (b–a)
**C18:2**	60.48	56.37	64.61	53.83	51.35	56.62	53.28	49.37	57.57	0.019 (c–a) 0.012 (b–a)

* Kruskal–Wallis test Bonferroni corrected.

## Data Availability

Data are available from the “corresponding authors” (e-mail: oliadmy@gmail.com (O.D.); eleftherios.panteris@gmail.com (E.P.); gkikae@auth.gr (H.G.)) upon reasonable request and with permission of AHEPA University Hospital.

## Supplementary Materials

The following are available online at https://www.mdpi.com/article/10.3390/biom12030354/s1, Supplementary Table S1. Median values (μg/L) of all measured acylcarnitines and 95% CIs for ACS and CCS patients; Supplementary Figure S1. (A) ACS vs. CCS, Log scaled Box plots of C8, C10, C16, C18.1, and C18.2 levels distribution (B) with their respective ROC Curves. Acylcarnitine C18.2 presented the highest, yet weak, discriminatory power AUC = 0.576 (95% CI 0.539–0.612, *p*  <  0.001); Supplementary Table S2. Diabetes Mellitus (DM) ROC areas for acylcarnitine C2, C3, C4, and C8; Supplementary Figure S2. (A) Log scaled bar graph for acylcarnitine median levels of DM and non-DM patients and (B) ROC curves for C2, C3, C4, and C8. Acylcarnitine C2 has the highest discriminatory power; Supplementary Table S3. Diabetes Mellitus (DM) ROC areas for acylcarnitines C2, C3, C4, and C8; Supplementary Table S4. Diabetes Mellitus (DM) ROC areas for acylcarnitines C8, C10, C16, C18.1, and C18.2 for ACS patients. Supplementary Figure S3. (A) Log scaled bar graphs for acylcarnitine median levels for both ACS and CCS, DM and non-DM patients. (B) ROC analysis of C8, C10, C16, C18.1, and C18.2 showed that for non-diabetics, AUCs were improved especially for C18.2. Supplementary Table S5. Acylcarnitine levels (μg/ L) for CAD groups. Supplementary Table S6. Acylcarnitines levels (μg/L) and SYNTAX Score groups; Supplementary Table S7. Acylcarnitine levels (μg/L) and SS metrics (heavy calcification and LVEF%); Supplementary Table S8. Linear regression for SYNTAX Score; Supplementary Table S9. Acylcarnitine levels (μg/L) comparison between kidney failure and non-kidney failure patients (GFR < 55); Supplementary Figure S4. Log10 scaled histogram of acylcarnitine levels comparison between kidney failure and non-kidney failure patients (GFR < 55).
