# Supplementary material for: Correlation of Serum Acylcarnitines with Clinical Presentation and Severity of Coronary Artery Disease
Source: Biomolecules. 2022 Feb 23;12(3):354. doi: 10.3390/biom12030354 (PMC8945505; doi:10.3390/biom12030354)
Supplement: Supplementary file 1 [file biomolecules-12-00354-s001.zip › biomolecules-1520405-supplementary.pdf]

Supplementary material

Correlation of Serum Acylcarnitines with clinical presentation and severity of Coronary Artery Disease

Olga Deda[†1,2*], Eleftherios Panteris[†1,2*], Thomas Meikopoulos[2,3], Olga Begou[2,3], Thomai Mouskeftara[1,2], Efstratios Karagiannidis[4], Andreas S. Papazoglou[4], Georgios Sianos[4], Georgios Theodoridis[2,3], Helen Gika[1,2*]

[1]Laboratory of Forensic Medicine and Toxicology, School of Medicine, Aristotle University of Thessaloniki, 54124, Thessaloniki, Greece

oliadmy@gmail.com, eleftherios.panteris@gmail.com, gkikae@auth.gr

[2]Biomic_AUTh, CIRI-AUTH Center for Interdisciplinary Research and Innovation Aristotle University of Thessaloniki, 57001, Thessaloniki, Greece

thomas_meik@hotmail.com, olina_18@hotmail.com, mouskeftara32@gmail.com, gtheodor@chem.auth.gr

[3]Laboratory of Analytical Chemistry, Department of Chemistry, Aristotle University of Thessaloniki, Thessaloniki, Greece

[4]First Department of Cardiology, AHEPA University Hospital, Aristotle University of Thessaloniki, St. Kiriakidi 1, 54636, Thessaloniki, Greece

stratoskarag@gmail.com, anpapazoglou@yahoo.com, gsianos@auth.gr

[†]Equal contribution

*Correspondence: oliadmy@gmail.com (O.D.); eleftherios.panteris@gmail.com (E.P), gkikae@auth.gr (H.G.)

Table S1. Median values (μg/L) of all measured acylcarnitines and 95% CIs for ACS and CCS patients. Statistically significant analogues are shown in bold.

	CCS			ACS			Mann-Whitney U test
	Median	**↓95.0% CIs**	**↑95.0% CIs**	Median	**↓95.0% CIs**	**↑95.0% CIs**	p
C2	2,970.44	2,846.84	3,111.70	2,933.31	2,761.05	3,059.40	0.949
C3	176.75	169.34	185.38	175.42	166.99	181.98	0.608
C4	38.65	36.74	41.17	38.76	36.31	40.90	0.736
C5	25.71	24.95	27.50	26.41	25.16	28.32	0.251
C6	29.98	28.79	31.46	28.51	26.86	29.77	0.265
C8	**63.06**	**58.68**	**68.55**	**54.75**	**51.21**	**57.68**	**0.012**
C10	**106.12**	**96.74**	**116.37**	**88.51**	**83.51**	**93.92**	**0.007**
C12	29.89	28.30	31.39	27.76	25.20	29.04	0.072
C14	19.23	18.51	19.79	17.98	17.25	19.01	0.061
C16	**63.21**	**60.85**	**65.63**	**59.97**	**57.66**	**61.97**	**0.018**
C18	19.01	18.35	19.52	18.27	17.63	18.84	0.060
C18:1	**92.54**	**88.15**	**97.76**	**84.43**	**80.05**	**89.91**	**0.011**
C18:2	**60.22**	**57.89**	**63.29**	**51.89**	**50.44**	**54.67**	**<0.001**

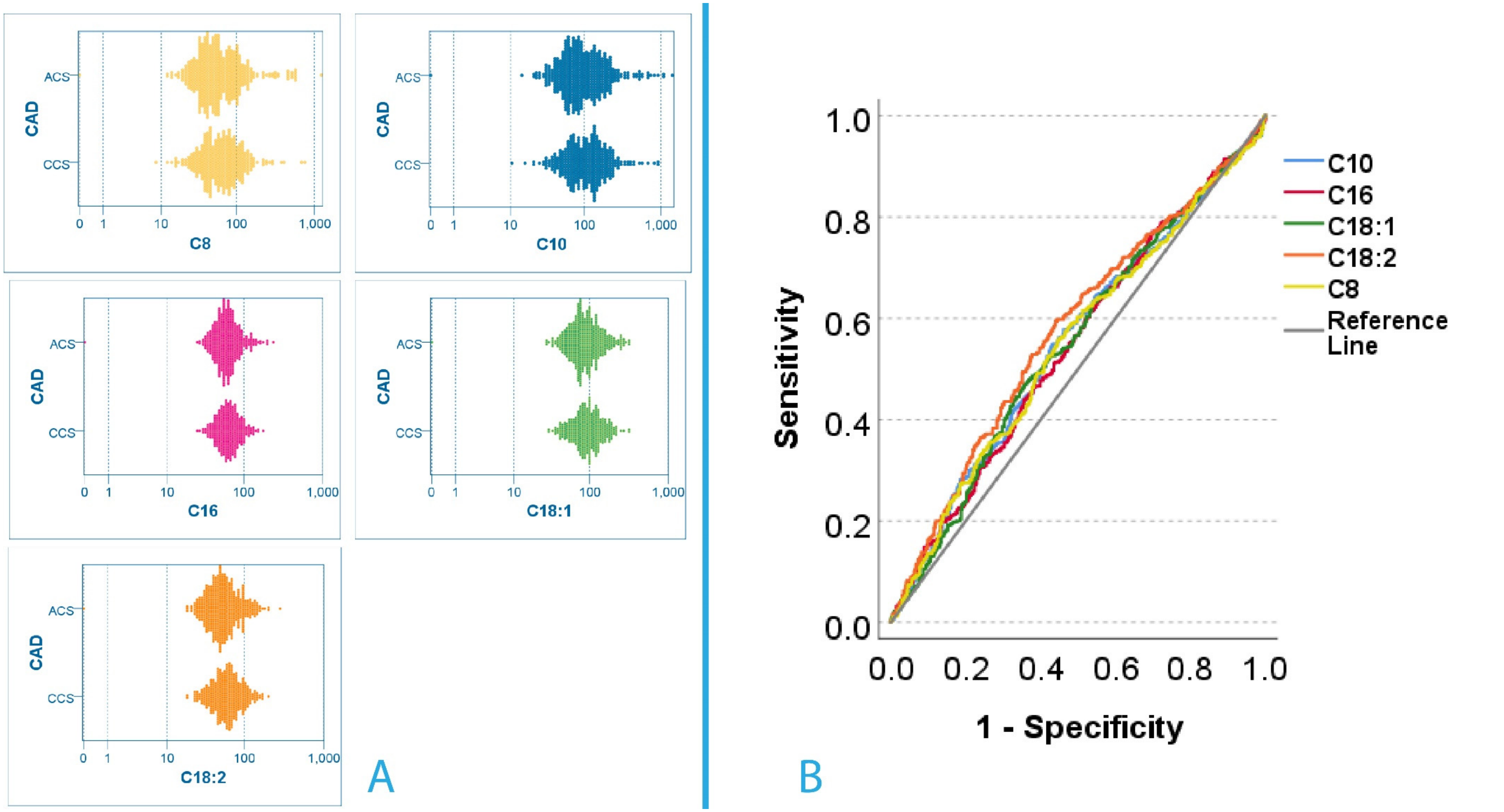

Figure S1. A) ACS *vs* CCS, log scaled box-plots of C8, C10, C16, C18.1 and C18.2 levels distribution and B) their respective ROC curves. Acylcarnitine C18.2 presented the highest, yet weak, discriminatory power AUC=0.576 (95% CI 0.539–0.612, $p < 0.001$).

Table S2. Diabetes Mellitus (DM) ROC areas for acylcarnitine C2, C3, C4 and C8.

Area Under the ROC Curve					
Test Result Variable(s)	Area	Std. Error[a]	Asymptotic Sig.[b]	Asymptotic 95% Confidence Intervals	
				**↓95.0% CIs**	**↑95.0% CIs**
**C2**	**0.591**	**0.019**	**0.000**	**0.553**	**0.629**
C3	0.576	0.019	0.000	0.538	0.614
C4	0.544	0.020	0.029	0.505	0.583
C8	0.541	0.020	0.040	0.502	0.580
a. Under the nonparametric assumption					
b. Null hypothesis: true area = 0.5					

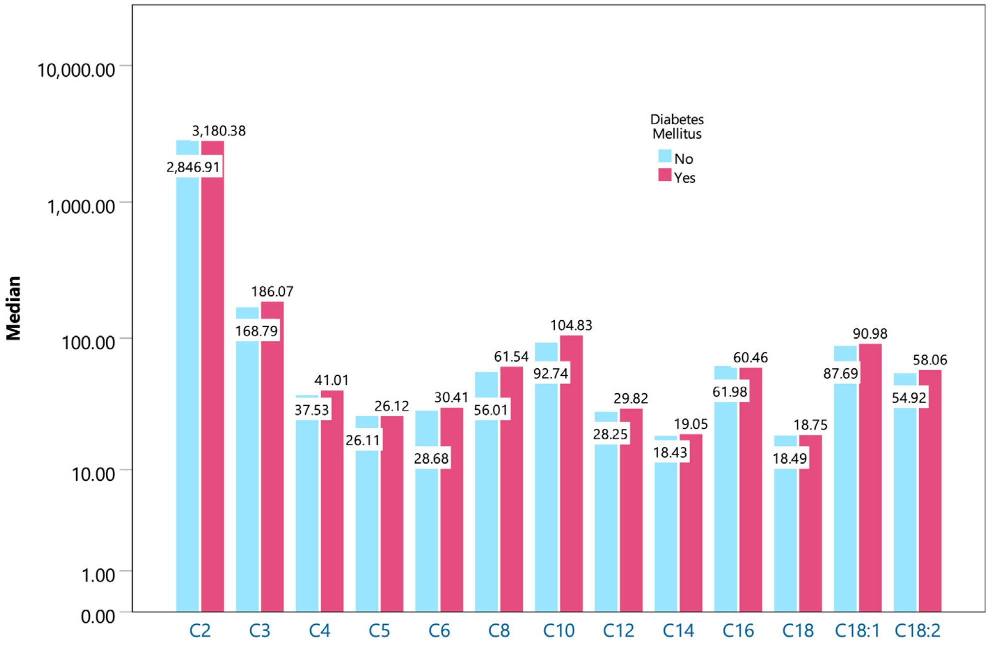

A

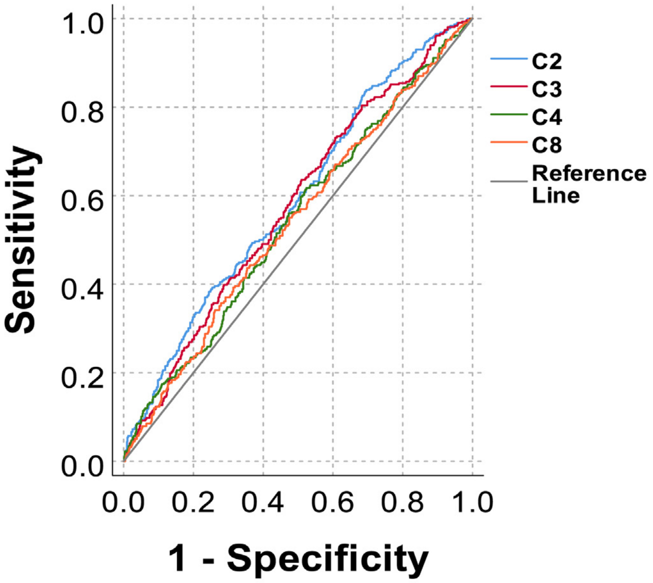

B

Figure S2. A) Log scaled bar graph for acylcarnitine median levels of DM and non-DM patients and B) ROC curves for C2, C3, C4, C8. Acylcarnitine C2 has the highest discriminatory power.

Table S3. Diabetes Mellitus (DM) ROC areas for acylcarnitines C2, C3, C4 and C8.

**Area Under the ROC Curve**					
Test Result Variable(s)	Area	Std. Error[a]	Asymptotic Sig.[b]	Asymptotic 95% Confidence Intervals	
				**↓95.0% CIs**	**↑95.0% CIs**
C2	0.591	0.019	0.000	0.553	0.629
C3	0.576	0.019	0.000	0.538	0.614
C4	0.544	0.020	0.029	0.505	0.583
C8	0.541	0.020	0.040	0.502	0.580
a. Under the nonparametric assumption					
b. Null hypothesis: true area = 0.5					

Table S4. Diabetes Mellitus (DM) ROC areas for acylcarnitines C8, C10, C16, C18.1, C18.2 for ACS patients.

Area Under the ROC Curve						
Diabetes Mellitus	Test Result Variable(s)	Area	Std. Error[a]	Asymptotic Sig.[b]	Asymptotic 95% Confidence Intervals	
					**↓95.0% Cis**	**↑95.0% Cis**
No	C8	0.561	0.023	0.007	0.517	0.606
	C10	0.567	0.023	0.003	0.522	0.612
	C16	0.559	0.023	0.011	0.514	0.604
	C18:1	0.568	0.023	0.003	0.523	0.613
	**C18:2**	**0.593**	**0.023**	**0.000**	**0.549**	**0.638**
Yes	C8	0.518	0.033	0.572	0.455	0.582
	C10	0.517	0.033	0.611	0.453	0.581
	C16	0.518	0.033	0.577	0.454	0.582
	C18:1	0.508	0.033	0.813	0.444	0.572
	C18:2	0.542	0.033	0.201	0.478	0.606
a. Under the nonparametric assumption						
b. Null hypothesis: true area = 0.5						

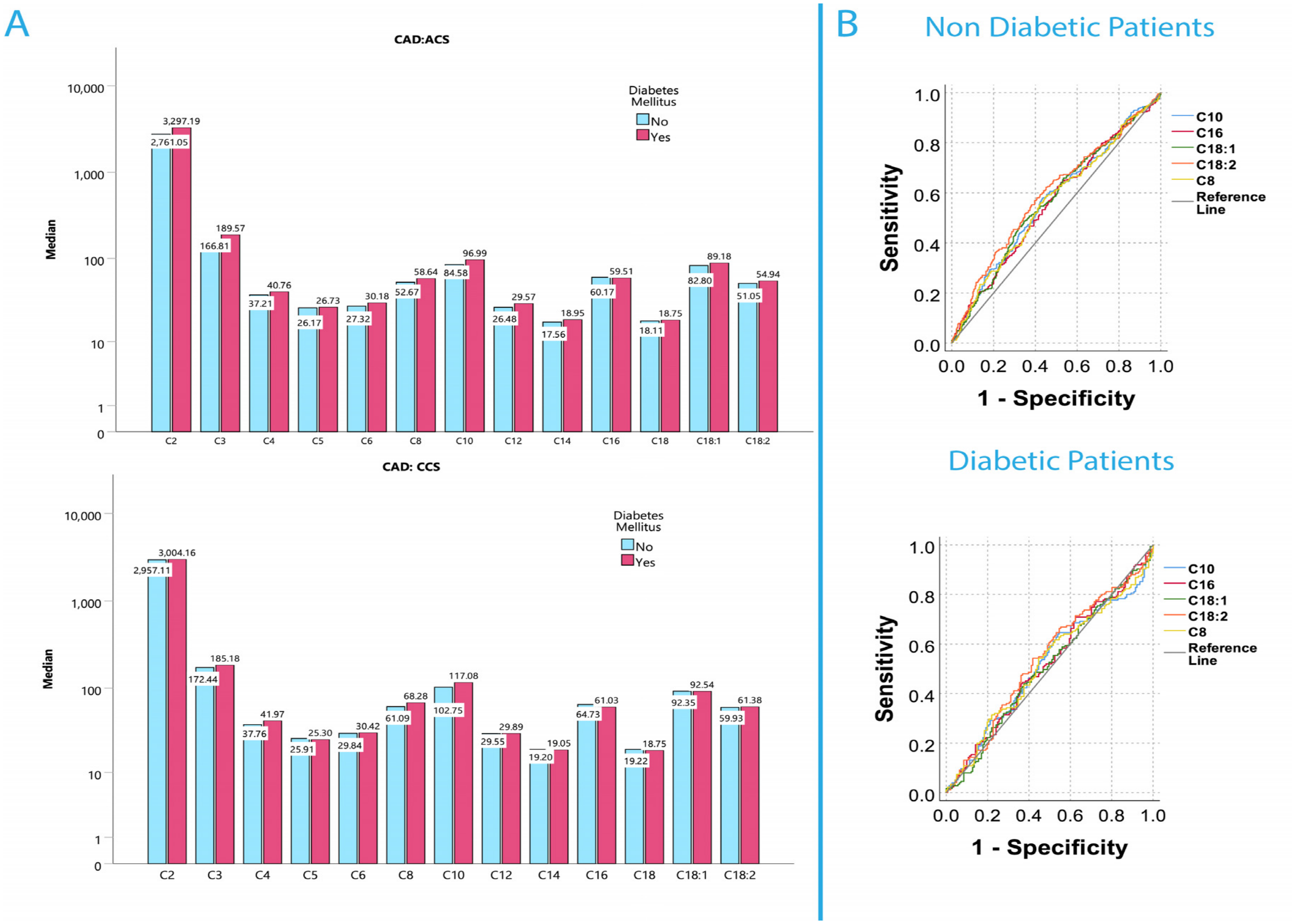

Figure S3. A) Log scaled bar graphs for acylcarnitine median levels for both ACS and CCS, DM and non-DM patients and B) ROC analysis of C8, C10, C16, C18.1 and C18.2 showed that for non-diabetics, AUCs were improved especially for C18.2.

Table S5. Acylarnitine levels (μg/ L) for CAD groups

CAD Groups					Kruskal Wallis
		Median	**↓95.0% CIs**	**↑95.0% CIs**	p value*
C2	NSTEMI	2,906.50	2,649.44	3,154.25	0.219
	STEMI	2,770.56	2,539.30	3,045.37	
	UA	3,147.27	2,839.91	3,452.40	
	SA	2,969.54	2,846.84	3,111.70	
C3	NSTEMI	168.35	151.99	182.39	0.824
	STEMI	173.55	157.15	191.74	
	UA	181.15	167.93	196.96	
	SA	176.80	169.84	185.38	
C4	NSTEMI	39.92	36.20	44.81	0.782
	STEMI	38.10	35.60	42.41	
	UA	37.99	34.83	43.02	
	SA	38.62	36.74	40.81	
C5	NSTEMI	24.79	23.46	28.80	**0.026**
	**STEMI**	**29.08**	**26.36**	**30.73**	
	**UA**	**25.13**	**22.86**	**27.70**	
	SA	25.72	24.95	27.50	
C6	NSTEMI	27.46	25.83	31.00	0.329
	STEMI	27.58	25.65	29.10	
	UA	30.69	27.77	33.83	
	SA	29.98	28.87	31.46	
C8	NSTEMI	54.66	49.00	60.09	0.053
	STEMI	52.99	49.11	58.13	
	UA	56.60	51.90	66.67	
	SA	63.06	58.68	68.55	
C10	NSTEMI	89.88	78.25	105.42	**0.019**
	**STEMI**	**86.53**	**79.36**	**94.45**	
	**UA**	**91.50**	**83.49**	**110.42**	
	SA	106.09	96.74	116.37	
C12	NSTEMI	26.54	23.70	30.20	0.377
	STEMI	27.96	25.11	29.83	
	UA	29.17	24.86	31.54	
	SA	29.88	28.26	31.39	
C14	NSTEMI	17.51	16.27	19.55	0.301
	STEMI	17.78	17.04	18.86	
	UA	19.28	17.52	20.69	
	SA	19.17	18.43	19.79	
C16	NSTEMI	58.38	55.03	63.64	**0.012**
	**STEMI**	**58.29**	**55.82**	**60.89**	
	**SA**	**63.21**	**60.85**	**65.63**	
	UA	62.90	60.82	66.52	
C18	NSTEMI	17.35	16.09	18.35	0.137
	STEMI	18.63	17.76	19.54	
	UA	18.63	17.49	19.29	
	SA	19.01	18.45	19.59	
C18:1	NSTEMI	85.82	78.64	94.91	**0.013**
	**STEMI**	**82.80**	**76.43**	**88.61**	
	**SA**	**92.53**	**88.15**	**97.76**	
	UA	91.30	79.31	97.06	
C18:2	NSTEMI	54.86	50.74	59.54	**<0.001**
	**STEMI**	**50.26**	**47.34**	**52.41**	
	**SA**	**60.21**	**57.89**	**63.29**	
	UA	53.75	50.00	60.48	

* Bonferroni corrected

Table S6. Acylcarnitines levels (µg/L) and Syntax Score groups.

Syntax Groups		Median	↓95.0% CIs	↑95.0% CIs	Kruskal Wallis (Pair) p value
C2	0 (a)	3,013	2,848.26	3,146.54	NS
	1-22 (b)	2,841	2,652.45	3,047.79	NS
	>22 (c)	2,951.55	2,798.57	3,325.86	NS
C3	0 (a)	171.49	162.86	179.56	NS
	1-22 (b)	175.68	167.86	184.94	NS
	>22 (c)	186.56	170.45	209.04	NS
**C4**	0 (a)	36.96	34.21	40.46	(a-c) 0.002
	1-22 (b)	37.95	35.60	40.18	(b-c) 0.005
	>22 (c)	45.16	38.94	49.61	
**C5**	0 (a)	25.25	23.99	26.36	(a-c) 0.024
	1-22 (b)	26.41	24.95	28.63	
	>22 (c)	27.82	25.34	30.79	
C6	0 (a)	29.81	28.07	33.04	NS
	1-22 (b)	28.89	27.63	30.08	NS
	>22 (c)	28.88	27.10	31.34	NS
C8	0 (a)	60.09	56.03	66.36	NS
	1-22 (b)	57.65	53.41	61.45	NS
	>22 (c)	55.13	49.58	62.70	NS
C10	0 (a)	99.63	90.03	109.52	NS
	1-22 (b)	94.48	86.47	101.15	NS
	>22 (c)	92.32	80.54	104.81	NS
C12	0 (a)	29.55	27.41	31.17	NS
	1-22 (b)	28.53	26.63	30.33	NS
	>22 (c)	27.73	25.11	29.71	NS
C14	0 (a)	19.24	18.12	20.36	NS
	1-22 (b)	18.54	17.59	19.27	NS
	>22 (c)	18.21	17.06	19.69	NS
**C16**	0 (a)	65.18	62.57	67.90	(c-a) 0.031
	1-22 (b)	60.28	57.95	62.48	(b-a) 0.044
	>22 (c)	59.27	56.28	61.94	NS
C18	0 (a)	19.11	18.27	20.08	NS
	1-22 (b)	18.39	17.70	19.03	NS
	>22 (c)	18.18	17.50	19.08	NS
C18:1	0 (a)	91.70	85.27	98.82	NS
	1-22 (b)	88.03	82.80	92.55	NS
	>22 (c)	87.63	80.05	93.49	NS
**C18:2**	0 (a)	60.48	56.37	64.61	(c-a) 0.019
	1-22 (b)	53.83	51.35	56.62	(b-a) 0.012
	>22 (c)	53.28	49.37	57.57	NS

NS= non-significant, $p > 0.05$

Table S7. Acylcarnitine levels (μg/L) and SS metrics (Heavy calcification and LVEF%).

Heavy calcification		Median	↓95.0% CIs	↑95.0% CIs	Mann-Whitney P value
C2	No	2,901.34	2,728.44	3,017.08	**0.002**
	Yes	3,121.71	2,933.37	3,325.86	
C3	No	169.84	165.03	176.21	**<0.001**
	Yes	200.02	183.58	213.75	
C4	No	36.74	35.23	38.67	**<0.001**
	Yes	45.77	41.23	50.19	
C5	No	25.55	24.85	26.69	**0.002**
	Yes	29.08	26.65	30.90	
C6	No	28.78	27.65	29.84	**0.006**
	Yes	30.33	28.66	32.17	
C8	No	57.71	54.65	60.51	0.104
	Yes	58.01	54.41	67.42	
C10	No	93.40	86.47	100.78	0.149
	Yes	97.62	92.32	113.59	
C12	No	28.70	27.33	29.95	0.468
	Yes	28.58	26.74	30.72	
C14	No	18.64	17.88	19.28	0.651
	Yes	18.96	17.28	20.02	
C16	No	62.34	60.31	64.11	0.365
	Yes	58.64	55.76	61.94	
C18	No	18.76	18.24	19.19	0.207
	Yes	18.10	17.38	19.02	
C18:1	No	87.65	83.89	92.22	0.327
	Yes	91.51	85.58	97.33	
C18:2	No	55.82	54.11	58.50	0.937
	Yes	54.23	51.18	59.94	
**LVEF groups**		**Median**	**↓95.0% CIs**	**↑95.0% CIs**	**Kruskal Wallis (Pair) P value**
C2	<40 (a)	3,255.34	3,022.59	4,185.32	(b-c) 0.006
	40-49 (b)	2,639.96	2,416.06	3,044.11	(a-c) 0.002
	>50 (c)	2,905.99	2,700.97	3,021.90	
C3	<40 (a)	206.90	175.46	243.44	(a-c) 0.001
	40-49 (b)	174.62	157.34	206.79	
	>50 (c)	171.25	165.03	178.98	(b-c) 0.006
C4	<40 (a)	52.70	39.71	58.60	(a-c) 0.001
	40-49 (b)	41.15	36.35	49.85	
	>50 (c)	36.60	34.83	38.94	
C5	<40 (a)	29.47	25.09	37.60	(b-c) 0.025
	40-49 (b)	28.92	25.43	31.66	(a-c) 0.006
	>50 (c)	25.13	24.02	26.14	
C6	<40 (a)	34.14	30.30	43.81	(a-b) 0.020
	40-49 (b)	29.25	26.86	32.84	(a-c) <0.001
	>50 (c)	27.81	26.62	29.26	
C8	<40 (a)	59.00	50.92	73.91	NS
	40-49 (b)	58.15	51.21	74.69	NS
	>50 (c)	55.22	51.17	59.64	NS
C10	<40 (a)	95.87	80.14	121.95	NS
	40-49 (b)	98.47	86.44	120.75	NS
	>50 (c)	92.34	84.57	98.89	NS
C12	<40 (a)	29.84	24.63	35.27	NS
	40-49 (b)	28.44	24.46	32.16	NS
	>50 (c)	27.55	26.17	29.02	NS
C14	<40 (a)	19.81	17.72	21.54	NS
	40-49 (b)	18.05	16.75	19.55	NS
	>50 (c)	18.39	17.43	19.14	NS
C16	<40 (a)	57.57	54.59	68.83	NS
	40-49 (b)	59.20	56.10	63.27	NS
	>50 (c)	61.33	58.85	63.55	NS
C18	<40 (a)	17.92	17.10	19.31	NS
	40-49 (b)	19.03	17.06	20.45	NS
	>50 (c)	18.22	17.63	18.76	NS
C18:1	<40 (a)	87.09	76.43	106.84	NS
	40-49 (b)	84.36	76.22	93.48	NS
	>50 (c)	86.04	81.90	90.19	NS
C18:2	<40 (a)	56.04	53.04	63.51	NS
	40-49 (b)	55.39	50.31	60.64	NS
	>50 (c)	53.63	50.74	57.07	NS

Table S8. Linear regression for Syntax Score.

Model Summary[b]

Model	R	R Square	Adjusted R Square	Std. Error of the Estimate	Change Statistics					Durbin-Watson
					R Square Change	F Change	df1	df2	Sig. F Change	
1	0.404[a]	0.164	0.157	12.3377	0.164	25.773	7	923	0.000	1.566

a. Predictors: (Constant), STATIN, Sex, Ratio C4/C18.2, CAD Groups, Diabetes Mellitus, Age, Smoking

b. Dependent Variable: Syntax Score

Bootstrap for Model Summary

Model	Durbin-Watson	Bootstrap[a]			
		Bias	Std. Error	95% Confidence Interval	
				Lower	Upper
1	1.566	-0.534	0.063	0.906	1.152

a. Unless otherwise noted, bootstrap results are based on 1000 bootstrap samples

ANOVA[a]

Model		Sum of Squares	df	Mean Square	F	Sig.
1	Regression	27,462.184	7	3,923.169	25.773	0.000[b]
	Residual	140,499.070	923	152.220		
	Total	167,961.254	930			

a. Dependent Variable: Syntax Score

b. Predictors: (Constant), STATIN, Sex, Ratio C4/C18.2, CAD Groups, Diabetes Mellitus, Age, Smoking

Coefficients[a]

Model		Unstandardized Coefficients		Standardized Coefficients	t	Sig.	95.0% Confidence Interval for B	
		B	Std. Error	Beta			Lower Bound	Upper Bound
1	(Constant)	5.432	2.85		1.904	0.057	-0.168	11.032
	Sex	2.968	0.950	0.098	3.126	0.002	1.105	4.832
	Diabetes Mellitus	2.056	0.438	0.145	4.689	0.000	1.195	2.916
	Smoking	0.368	0.897	0.014	0.411	0.681	-1.392	2.129
	Age	0.156	0.036	0.143	4.356	0.000	0.086	0.226
	Ratio C4/C18.2	2.010	0.473	0.130	4.250	0.000	1.082	2.938
	CAD Groups	-3.091	0.357	-0.269	-8.657	0.000	-3.791	-2.390
	STATIN	2.192	0.825	0.082	2.656	0.008	0.572	3.811

a. Dependent Variable: Syntax Score

Bootstrap for Coefficients

Model		B	Bootstrap[a]				
			Bias	Std. Error	Sig. (2-tailed)	95% Confidence Interval	
						Lower	Upper
1	(Constant)	5.432	0.009	2.714	0.038	0.308	10.510
	Sex	2.968	-0.004	0.988	0.005	1.048	4.883
	Diabetes Mellitus	2.056	0.002	0.470	0.001	1.150	2.978
	Smoking	0.368	-0.062	0.848	0.651	-1.428	1.985
	Age	0.156	-0.001	0.035	0.001	0.087	0.219
	Ratio C4/C18.2	2.010	0.124	0.613	0.002	1.159	3.587
	CAD Groups	-3.091	0.007	0.370	0.001	-3.803	-2.334
	STATIN	2.192	-0.007	0.849	0.011	0.489	3.870

a. Unless otherwise noted, bootstrap results are based on 1000 bootstrap samples

Table S9. Acylcarnitine levels (μg/L) comparison between CKD and non-CKD patients

	CKD patients (N = 127) vs Non CKDN = 818).						
	Non CKD			CKD			Mann Whitney test
	Median	↓95.0% CIs	↑95.0% CIs	Median	↓95.0% CIs	↑95.0% CIs	p value
C2	2,800.68	2,654.27	2,924.54	4,186.94	3,926.20	4,584.89	<0.001
C3	169.97	164.87	175.74	232.15	209.04	250.07	<0.001
C4	36.74	35.30	38.39	58.42	53.10	67.68	<0.001
C5	25.55	24.82	26.60	35.92	30.03	40.57	<0.001
C6	27.95	26.99	28.87	43.68	39.96	49.39	<0.001
C8	54.41	51.75	56.90	92.21	83.67	100.58	<0.001
C10	89.64	83.69	94.45	153.27	137.40	171.24	<0.001
C12	27.40	26.10	28.60	35.56	32.81	45.20	<0.001
C14	18.15	17.41	18.85	21.87	20.46	23.82	<0.001
C16	60.43	58.47	62.35	66.04	61.84	73.46	0.003
C18	18.35	17.86	18.86	19.24	18.49	21.36	<0.001
C18:1	87.22	83.38	90.38	97.50	91.44	105.93	<0.001
C18:2	54.92	52.75	57.07	60.04	54.99	65.12	0.016

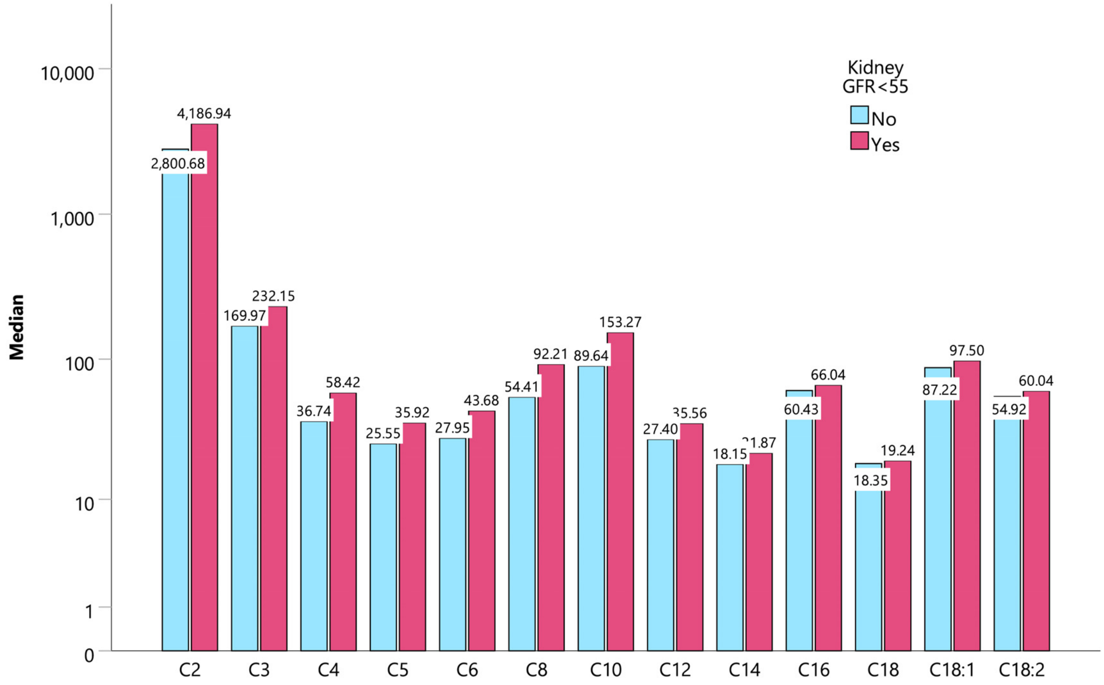

Figure S4.) Log scaled bar graphs of acylcarnitine levels comparison between kidney failure and non-kidney failure patients (GFR<55).
